# Supplementary material for: Observation of strong nonlinear interactions in parametric down-conversion of X-rays into ultraviolet radiation
Source: Nat Commun. 2019 Dec 12;10:5673. doi: 10.1038/s41467-019-13629-w (PMC6908627; doi:10.1038/s41467-019-13629-w)
Supplement: Supplementary file 1 — Supplementary Information [file 41467_2019_13629_MOESM1_ESM.pdf]

Supplementary Material for "Observation of strong nonlinear interactions in parametric down-conversion of x-rays into ultraviolet radiation"

Authors: S. Sofer[1*], O. Sefi[1*], E. Strizhevsky[1], H. Aknin[1], S.P. Collins[2], G. Nisbet [2], B. Detlefs[3], Ch.J. Sahle[3], and S. Shwartz[1]

*S. Sofer and O. Sefi contributed equally to this work.

Affiliations:

[1]Physics Department and Institute of Nanotechnology, Bar-Ilan University, Ramat Gan, 52900 Israel

[2] Diamond Light Source, Harwell Science and Innovation Campus, Didcot OX11 0DE, United Kingdom

[3] ESRF – The European Synchrotron, CS 40220, 38043 Grenoble Cedex 9, France.

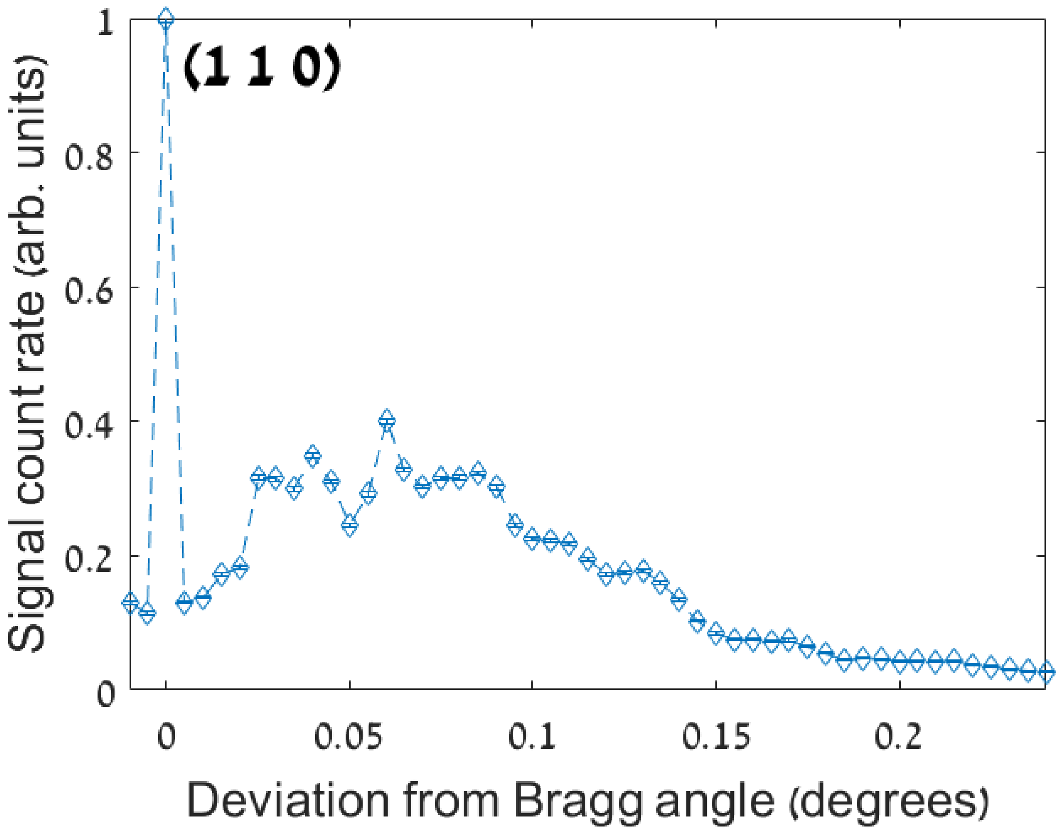

Supplementary Figure 1: **$LiNbO_3$ rocking curve for the (1 1 0) atomic planes at idler energy of 5 eV.** The PDC signal is the broad peak whereas the narrow peak on the left is the residual elastic scattering. The angle of the pump wave vector at the peak of the rocking curve deviates by 0.06 degrees from the measured Bragg angle and the offset of the signal wave vector from the measured Bragg angle is 0.0225 degrees. The vertical error bars indicate the counting statistics.

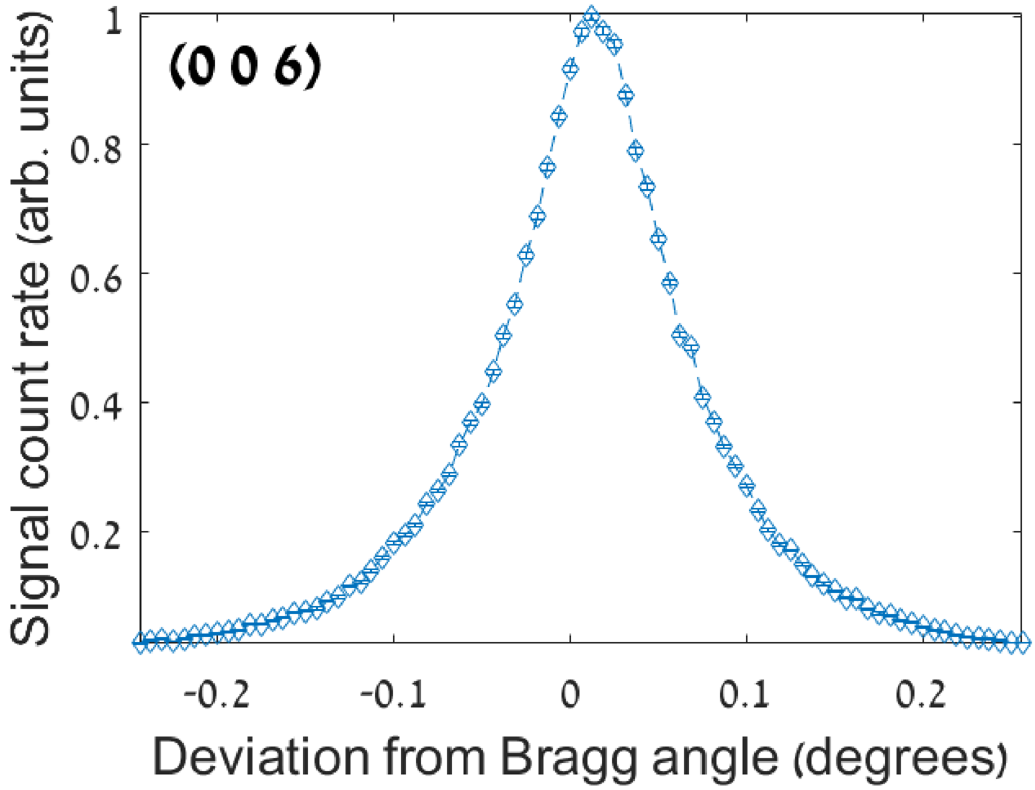

Supplementary Figure 2: **$LiNbO_3$ rocking curve for the (0 0 6) atomic planes at idler energy of 5 eV.** The angle of the pump wave vector at the peak of the rocking curve deviates by 0.006 degrees from the measured Bragg angle and the offset of the signal wave vector from the measured Bragg angle is 0.0225 degrees. The vertical error bars indicate the counting statistics.

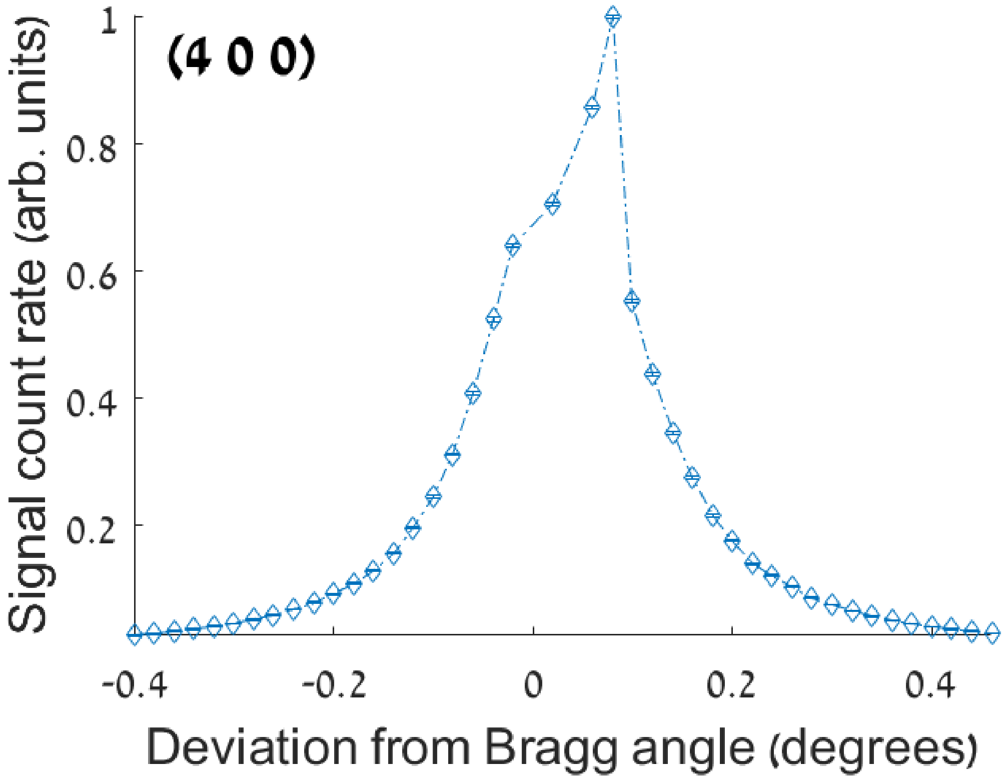

Supplementary Figure 3: **GaAs rocking curve for the (4 0 0) atomic planes at idler energy of 20 eV.** The angle of the pump wave vector at the peak of the rocking curve deviates by 0.08 degrees from the measured Bragg angle and the offset of the signal wave vector from the Bragg angle is -0.01 degrees. The vertical error bars indicate the counting statistics.

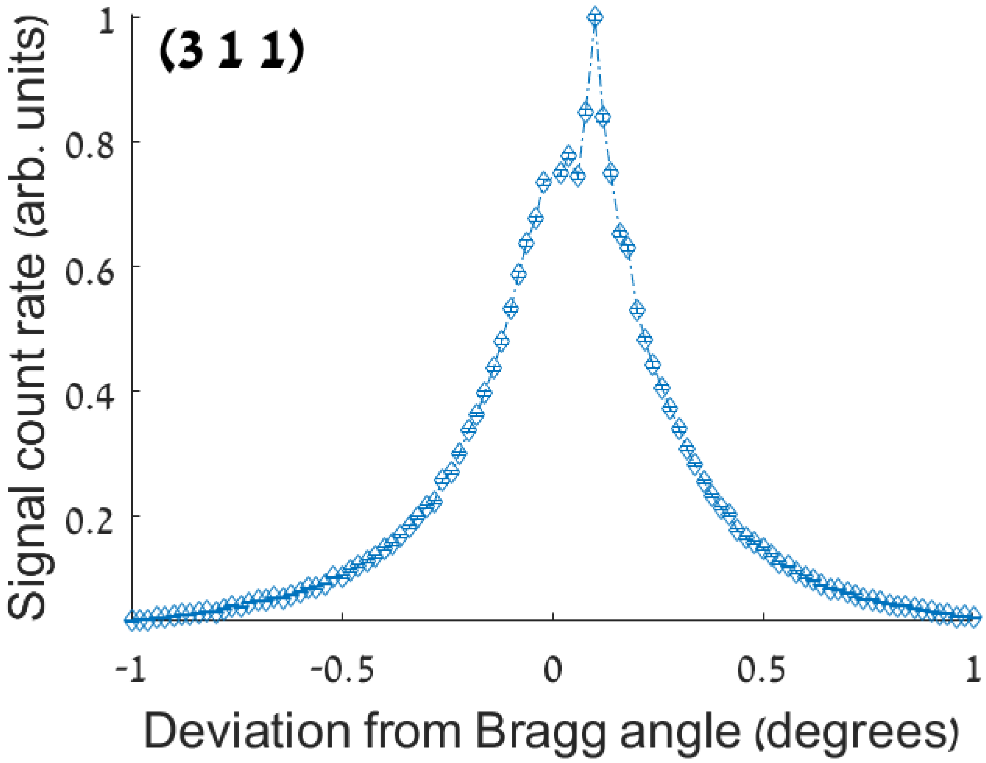

Supplementary Figure 4: **GaAs rocking curve for the (3 1 1) atomic planes at idler energy of 20 eV.** The angle of the pump wave vector at the peak of the rocking curve deviates by 0.14 degrees from the Bragg angle and the offset of the signal wave vector from the Bragg angle is -0.023 degrees. The vertical error bars indicate the counting statistics.

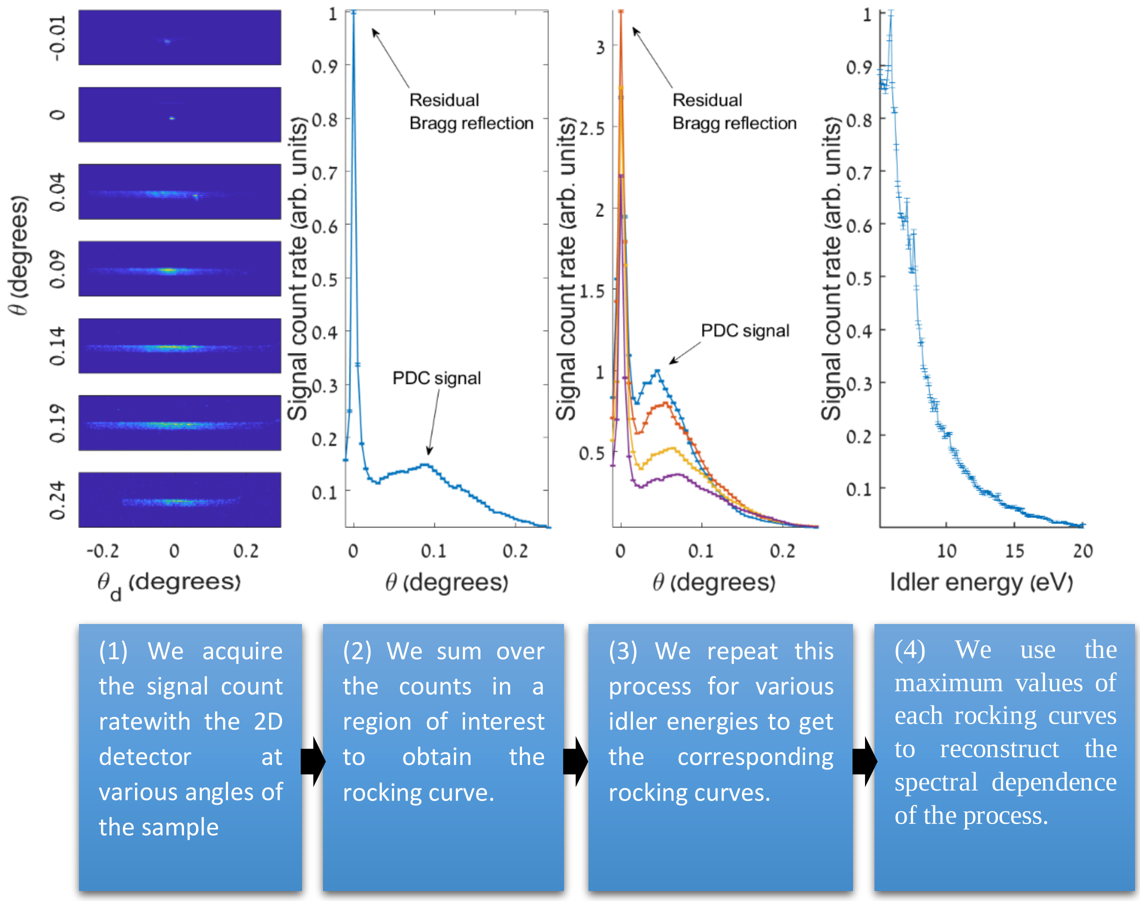

Supplementary Figure 5: **Scheme of the data analysis procedure**. θ_d is the deviation of the signal from the Bragg angle and θ is the deviation of the crystal angle from the Bragg angle. The vertical error bars indicate the counting statistics.

Supplementary Note 1 – Rocking curves

We provide further description on our procedures for the data analysis and for the validation of the measurements of the PDC signal. First, we show several examples of the measured rocking curves that show the agreement with the calculated phase matching angles and nonlinearity, thus constitute conclusive evidence that the measured signal is indeed PDC. We recall that the angular dependence of the PDC efficiency is determined by the phase matching condition and by the nonlinearity. Thus, we expect that the maximal efficiency of the effect will be observed near the phase matching angles and when the nonlinearity is the largest. The equation that describes the phase matching is $\mathbf{k}_\mathrm{p} + \mathbf{G} = \mathbf{k}_\mathrm{s} + \mathbf{k}_\mathrm{i}$. In order to estimate the shift from the phase matching condition, we use the phase mismatch, which is defined as $\Delta k_z L$ where Δk_z is the deviation from the phase matching condition in the propagation direction and L is the length of the crystal, which is defined by the shortest absorption length. We use the wave vectors at the peak of the rocking curve to evaluate the phase mismatch. In the case of PDC of X-rays into UV radiation, the short length is the absorption length of the UV wavelength. The phase mismatch describes the dependence of the efficiency of the PDC process on the deviation from the exact phase matching condition.

We start by showing the rocking curves of $LiNbO_3$ for the (1 1 0) atomic planes at idler energy of 5 eV in supplementary figure 1. The calculated mismatch is -0.575, which is much smaller than π.

We continue with the rocking curve for the$LiNbO_3$ (0 0 6) atomic planes for idler energy of 5 eV in supplementary figure 2. The calculated mismatch is -0.9789, which is smaller than π.

Next, we show examples for rocking curves of GaAs. Supplementary figure 3 shows the rocking curve measured for the (4 0 0) atomic planes for idler energy at 20 eV. The calculated phase mismatch is -1.221, which is also smaller than π.

We next show the rocking curve measured for the (3 1 1) atomic planes in supplementary figure 4. The calculated phase mismatch is -0.1, which is again much smaller than π.

We note that in addition to the uncertainties regarding the short UV absorption lengths, there are also experimental uncertainties that emerge from the bandwidth and the acceptance angles of the input monochromator and the analyzers. We estimate the overall energy precision to be 0.3 eV at ESRF and 1 eV at the Diamond Light Source.

Supplementary Note 2 – Detailed description of the analysis process

We describe our procedure for the reconstruction of the rocking curves and the spectra. As was mentioned in the main text, the spectra are reconstructed by finding the peaks of the rocking curves (the count rate of the signal as a function of the angle of the sample) at each of the photon energies of the idler (the longer wavelength photon). All rocking curves are generated by choosing a region of interest on the detector and summing over the counts within this region as a function of the detuning of angle of the sample from the Bragg angle. The regions of interests are chosen to be centered at about 20 pixels from the spot of the elastic scattering in the horizontal direction to block the strong signal from the residual elastic scattering. In the vertical direction they are centered around the peak. The sizes of the region of interest are about 50 pixels in the horizontal direction and 20 pixels in the vertical direction. The

peak of each rocking curve is used to reconstruct the spectral dependence of efficiency of the PDC for the chosen atomic planes. A scheme for the reconstruction of the spectrum for chosen atomic planes is shown in supplementary figure 5.

For the estimation of the efficiencies we take the peak of the rocking curve for the chosen atomic planes and sum over a region of interest that is defined by the full width at half the maximum of the signal PDC counts on the camera (while filtering any residual elastic scattering by removing them from the region of interest).
